# Maternal Gestational Diabetes Mellitus Modulates Adipose Tissue Remodeling and CTRP6 Expression in a Depot- and Sex-Specific Manner in Mouse Offspring

**DOI:** 10.3390/biomedicines14010224

**Published:** 2026-01-20

**Authors:** Xiaojing Wei, Jianan Jiang, Weijie Feng, Yutian Tan, Chao Sun, Jun Liu, Zhao Yang, Guiying Yang, Xiao Luo

**Affiliations:** 1 Department of Physiology and Pathophysiology, School of Basic Medical Sciences, Xi’an Jiaotong University Health Science Center, Xi’an Jiaotong University, Xi’an 710061, China; weixiaojing1022@xjtu.edu.cn (X.W.); jiangjianan0409@stu.xjtu.edu.cn (J.J.); fengwj@stu.xjtu.edu.cn (W.F.); tanyutian@stu.xjtu.edu.cn (Y.T.); 19845393766@163.com (C.S.); liu_i_jun@stu.xjtu.edu.cn (J.L.); 2 Institute of Neuroscience, Translational Medicine Institute, Xi’an Jiaotong University Health Science Center, Xi’an Jiaotong University, Xi’an 712046, China; 3 Department of Obstetrics and Gynecology, The First Affiliated Hospital of Xi’an Jiaotong University, Xi’an 710061, China; 18392886054@163.com (Z.Y.); yanggy0912@163.com (G.Y.)

**Keywords:** gestational diabetes mellitus, offspring, adipose tissue remodeling, CTRP6, high-fat diet

## Abstract

**Objective:** This study aimed to explore how maternal gestational diabetes mellitus (GDM) affects adipose tissue remodeling and the expression of C1q/TNF-related protein 6 (CTRP6) in offspring, with a focus on sex- and depot-specific differences. **Methods:** A GDM mouse model was established by feeding female C57BL/6J mice a high-fat diet (HFD) before and during pregnancy. Offspring were weaned onto standard chow or an HFD until 9 weeks of age. Metabolic phenotypes, adipose tissue morphology, and CTRP6 expression were assessed at weaning and adulthood. **Results:** GDM offspring exhibited increased adiposity and impaired glucose tolerance at weaning, with these effects persisting into adulthood in males. Maternal GDM reduced plasma CTRP6 levels in both sexes at weaning, but in adulthood, male GDM offspring maintained lower circulating CTRP6, while females on the chow diet showed elevated levels. Tissue-specific analysis revealed decreased CTRP6 expression in male interscapular brown adipose tissue (iBAT) and female visceral white adipose tissue (vWAT), accompanied by depot- and sex-specific changes in adiponectin signaling. **Conclusions:** Maternal GDM programs offspring’s metabolic phenotype and adipose tissue CTRP6 expression in a sex- and depot-specific manner, suggesting CTRP6 may serve as an early, sex-biased indicator of the intergenerational transmission of metabolic disease risk.

## 1. Introduction

Gestational diabetes mellitus (GDM) now complicates one in seven pregnancies worldwide and transmits a 2–8-fold higher risk of obesity and type 2 diabetes to the next generation [1,2,3,4,5]. Although this epidemiological link is well established [6,7,8,9,10], the mechanisms that underlie the immediate postnatal changes and later under obesogenic stress remain fragmentary. Adipose tissue is the most obvious candidate driver: human adipose tissue begins to form as early as the 14th week of gestation; it expands rapidly during early postnatal life, and its mode of expansion—hyperplasia versus hypertrophy—determines lifelong metabolic risk [11,12]. Growing evidence highlights that appropriate adipose tissue remodeling is essential for maintaining offspring energy homeostasis [13,14,15]. Whether GDM disturbs this developmental “remodeling” program in a depot- and sex-specific manner is still unknown.

C1q/TNF-related protein 6 (CTRP6), the closest adiponectin paralog, is a prime candidate mediator. This 240-aa secreted glycoprotein is abundantly expressed in adipose tissue, circulates as oligomers, and regulates glucose uptake, lipid oxidation, and inflammatory responses [16,17,18]. Clinically, circulating CTRP6 levels are increased in overweight or obese individuals and associated with insulin resistance parameters [19]. Whole-body or adipocyte-specific knockdown protects mice from diet-induced weight gain and insulin resistance, whereas transgenic overexpression blunts Akt phosphorylation and suppresses adipogenesis [18,20]. Critically, CTRP6 exhibits marked sexual dimorphism; levels are highest in the female placenta, endometrium, and visceral fat [21,22]. Furthermore, it regulates adipose tissue expansion and inflammation in mice by rapidly responding to acute nutrient changes [23]. This rapid, nutritionally driven regulation—combined with its sexually dimorphic expression pattern—suggests that CTRP6 may serve as an early molecular bridge that conveys maternal metabolic status to the developing fetal adipose tissue. We recently identified that CTRP6 is already dysregulated in the placenta and cord blood from human GDM pregnancies [24]. Yet, its longitudinal profile across the three major adipose depots—interscapular brown adipose tissue (iBAT), subcutaneous white adipose tissue (sWAT), and visceral white adipose tissue (vWAT)—during the transition from suckling to independent feeding has never been examined.

In the current study, we established a GDM mouse model and challenged the offspring with a high-fat diet from postnatal day 21 (PND21) to 9 weeks of age. By simultaneously quantifying CTRP6 expression, adipose morphology, and systemic metabolism in both sexes at PND21 and week 9, we aimed to determine (i) whether maternal GDM programs CTRP6 in a depot- and sex-specific manner, (ii) how post-weaning HFD challenge modifies this signature, and (iii) whether CTRP6 dynamics precede or parallel the metabolic disturbances characteristic of GDM offspring.

## 2. Materials and Methods

### 2.1. Animal Care

Five-week-old female C57BL/6J mice were obtained from the Medical Laboratory Animal Center of Xi’an Jiaotong University. Mice were housed in specific-pathogen-free (SPF) conditions (20 ± 2 °C, a 12h light-dark cycle) with ad libitum access to standard chow (Beijing Keao Xieli Feed, Beijing, China; 3.85 kcal/g; 10%, 70%, and 20% of calories from fat, carbohydrate, and protein, respectively) and tap water. A GDM mouse model was established as previously described [24]. Briefly, mice were fed an HFD for six weeks before mating and throughout pregnancy. After one week of acclimation, they were randomly assigned to two dietary groups: standard chow (NGT, *n* = 20) or HFD (GDM, *n* = 20) (Beijing Keao Xieli Feed, Beijing, China; the D12451 diet, 4.73 kcal/g; 45%, 35%, and 20% of calories from fat, carbohydrate, and protein, respectively). Following six weeks of dietary intervention, random blood glucose levels were measured to confirm no baseline differences between groups.

**Figure 1:**
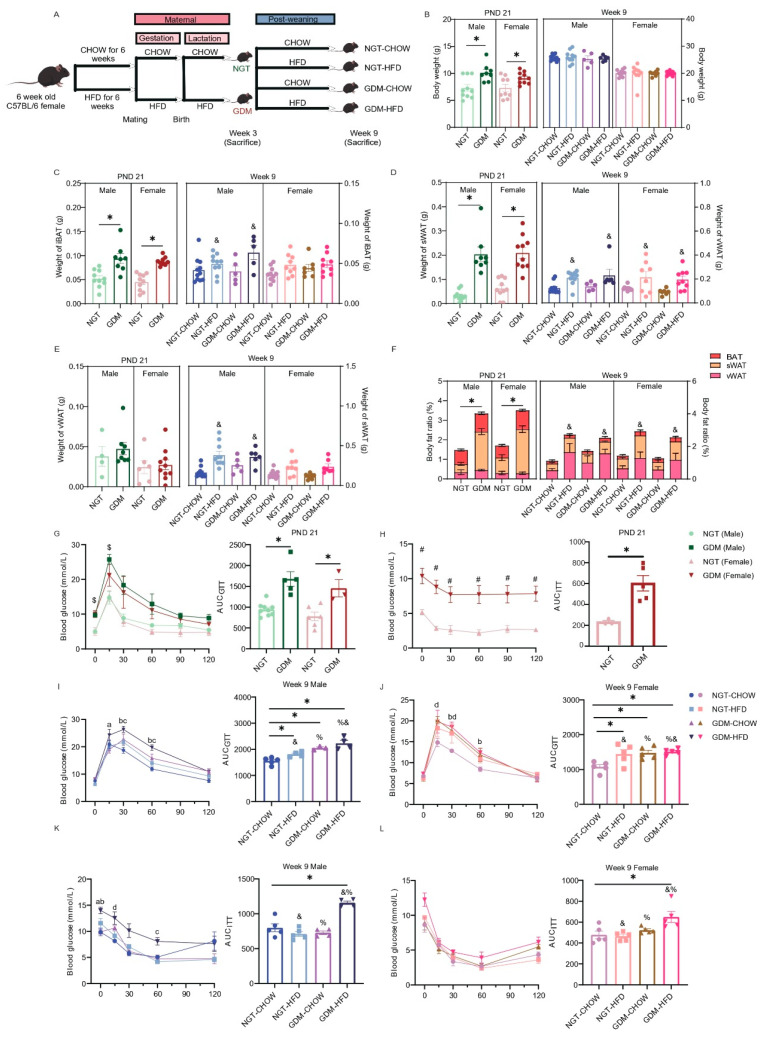
Metabolic phenotype of GDM offspring at weaning and young adulthood. (**A**) Timeline of animal experiments. Each group comprises one male and one female pup per litter; *n* represents the number of litters. No within-litter correlation was introduced. (**B**) Body weight. (**C**–**E**) Weight of brown adipose tissue (iBAT), subcutaneous white adipose tissue (sWAT), and visceral WAT (vWAT). (**F**) Body fat ratio (%), *n* = 5–10/group. (**G**,**H**) Glucose tolerance test (GTT), insulin tolerance test (ITT), and area under the curve (AUC) analysis at PND21, GTT: *n* = 3–9/group; ITT: *n* = 3–5/group. (**I**,**J**) GTT and AUC in male and female offspring at week 9, *n* = 5/group. (**K**,**L**) ITT and AUC in male and female offspring at week 9, *n* = 5/group. Data are presented as mean ± SEM. At PND21, differences between the two groups were evaluated with an unpaired two-tailed *t*-test. For the four offspring groups at 9 weeks, a two-way ANOVA was used; where interaction was significant, Bonferroni’s post hoc test was applied. %—main effect of GDM, *p* < 0.05 vs. maternal NGT groups; &—main effect of post-weaning HFD, *p* < 0.05 vs. post-weaning chow groups. * *p* < 0.05 for Bonferroni post-tests. (**G**–**L**) were analyzed by repeated measures ANOVA; $: *p* < 0.05 (NGT versus GDM in male); #: *p* < 0.05 (NGT versus GDM in female); a: *p* < 0.05 (GDM-chow versus GDM-HFD); b: *p* < 0.05 (NGT-chow versus GDM-HFD); c: *p* < 0.05 (NGT-HFD versus GDM-HFD); d: *p* < 0.05 (NGT-chow versus GDM-chow).

For breeding, two females and one male were housed together overnight, and the presence of a vaginal plug was designated as gestational day 0.5. The day of delivery was recorded as postnatal day (PND) 0. On postnatal day 1 (PND 1), each litter was standardized to six pups (three males and three females). The dams’ diets, established during gestation, were continued unchanged throughout the lactation period. At weaning (PND 21), one male and one female pup from each litter were euthanized. From the remaining pups (two males and two females per litter), one animal of each sex was randomly assigned to receive a standard chow diet, while the other was assigned to a high-fat diet (HFD). These post-weaning dietary regimens were maintained until the experimental endpoint at 9 weeks of age. Consequently, for each sex, four distinct experimental groups were generated based on the combination of maternal (NGT or GDM) and post-weaning (chow or HFD) diets: NGT-chow, NGT-HFD, GDM-chow, and GDM-HFD. The experimental timeline is illustrated in Figure 1A.

### 2.2. Glucose Tolerance Test (GTT) and Insulin Tolerance Test (ITT)

GTT and ITT were performed at both PND21 and 9 weeks. For the GTT, pups were fasted for 16 h and injected intraperitoneally with a saline glucose solution (2 g kg^−1^ body weight). Blood was collected from the tail vein, and glucose levels were detected by One Touch Sure Step Test Strips (Johnson & Johnson, New Brunswick, NJ, USA) before and at 15, 30, 60, 90, and 120 min after injection.

For the ITT, pups were intraperitoneally injected with a saline insulin (Humulin U-100, 0.75 IU kg^−1^ body weight) after a 4 h fast in weanlings or a 6 h fast in adults. Blood glucose was detected by One Touch Sure Step test strips (Johnson & Johnson, New Brunswick, NJ, USA) before and at 15, 30, 60, 90, and 120 min after insulin injection.

### 2.3. Sacrifice, Tissue Collection, and Histological Analysis

At PND21 and again at 9 weeks of age, one male and one female per litter were euthanized. After a 6 h fast, blood was collected by cardiac puncture under isoflurane anesthesia. The iBAT, inguinal sWAT, and gonadal vWAT were bilaterally dissected, weighed, and portions were snap-frozen at −80 °C for molecular analyses, while others were fixed in 4% paraformaldehyde (PFA) for haematoxylin–eosin (H&E) staining. All animal studies and procedures were approved by the Institutional Animal Care and Use Committee (IACUC) of Xi’an Jiaotong University (XJTU-2017-778).

Adipose tissue samples were fixed in 4% PFA overnight at 4 °C and then dehydrated in 70% ethanol. After paraffin embedding, 7-μm sections were cut and stained with H&E according to the standard protocol. Five random fields per mouse were imaged at 400× magnification with a BX53 microscope (Olympus, Tokyo, Japan); three mice per group were evaluated. Adipocyte size was quantified with ImageJ software (version 1.54g, National Institutes of Health, Bethesda, MD, USA).

### 2.4. Plasma Metabolic Profile

At PND 21 and again at week 9, a basic metabolic profile was obtained. After a 6 h fast, tail-blood glucose was measured with a Contour TS glucometer. Plasma harvested on the day of sacrifice was assayed for triglycerides (TG) and total cholesterol (CHO) with commercial kits (Nanjing Jiancheng, Nanjing, China). Plasma insulin and CTRP6 were quantified by ELISA (insulin, cat. H203-1-2; CTRP6, cat. H407-1; Nanjing Jiancheng) according to the manufacturer’s instructions. Insulin resistance was estimated with the homeostasis model assessment index: HOMA-IR = [fasting glucose (mmol L^−1^) × fasting insulin (µIU L^−1^)]/22.5.

### 2.5. Quantitative PCR Analysis

Total RNA was isolated with an RNA extraction kit (R0027, Beyotime, Beijing, China) according to the manufacturer’s instructions. The cDNA was synthesized with a RevertAid RT-PCR kit (Thermo Fisher Scientific, Waltham, MA, USA). Quantitative PCR was performed with gene-specific primers and SYBR Green I master mix (Takara, Dalian, China). Relative gene expression was calculated with the 2^−ΔΔCt^ method relative to the *gapdh* or *cyclophilin* housekeeping gene. All primers were purchased from AUGCT (Beijing, China); sequences are available upon request.

### 2.6. Western Blotting Analysis

Tissue samples were homogenized in RIPA lysis buffer (Life Technologies, Carlsbad, CA, USA) and lysed for 30 min at 4 °C. Protein concentration was determined with the Bradford assay (Bio-Rad, Hercules, CA, USA). Subsequently, 30 µg of protein per lane was resolved by 10% SDS-PAGE and transferred to polyvinylidene difluoride (PVDF) membrane (Millipore, Bedford, MA, USA). Membranes were blocked with 5% (*w*/*v*) skim milk in 0.1% TBST for 1 h at room temperature, followed by overnight incubation at 4 °C with primary antibodies: anti-CTRP6 (1:1000, cat. 36900, Abcam, Cambridge, UK) and anti-adiponectin (1:500, Cat No. 21613-1-AP, Proteintech, Wuhan, China). After washing, the membranes were incubated with horseradish peroxidase (HRP)-conjugated secondary antibodies for 1 h at room temperature. Signals were detected with Immobilon HRP substrate (Millipore) and visualized with a ChemiDoc Touch Imaging System (Bio-Rad, Hercules, CA, USA). Densitometry was performed using Image Lab software (version 5.2, Bio-Rad); data were normalized to total lane protein.

### 2.7. Statistical Analysis

All data are presented as mean ± SEM. Statistical analyses were performed using GraphPad Prism 9.0 (GraphPad Software Inc., San Diego, CA, USA). At PND21, differences between the two groups were evaluated with an unpaired two-tailed *t*-test. GTT and ITT data were analyzed by repeated-measures two-way ANOVA. For the four offspring groups at 9 weeks, a two-way ANOVA was used; where interaction was significant, Bonferroni’s post hoc test was applied. *p* < 0.05 was considered statistically significant.

## 3. Results

### 3.1. Metabolic Phenotype of GDM Offspring at Weaning and Young Adulthood

Experimental design is summarized in Figure 1A. As reported previously [24], GDM dams gained more weight, showed higher fasting glucose at GD 12.5 and GD 17.5, and exhibited elevated plasma insulin from GD 6.5 to GD 17.5, matching current criteria for a GDM mouse model [25]. Intraperitoneal GTT at GD 14.5 revealed higher glucose at 15 and 30 min and a larger AUC in GDM mice, despite comparable HOMA-IR between groups. Offspring metabolic phenotype was assessed at two developmental windows: weaning (PND21) and early adulthood (week 9). At PND21, pups born to GDM dams were significantly heavier than NGT controls of both sexes (*p* < 0.05; Figure 1B). This excess weight was accompanied by enlarged iBAT and sWAT depots and a higher whole-body fat percentage (*p* < 0.05; Figure 1C,D,F). By week 9, total body weight no longer differed among the four experimental groups in either sex (Figure 1B). However, two-way ANOVA of adipose depot masses revealed a main effect of post-weaning HFD, but not of maternal GDM exposure. Post-weaning HFD increased iBAT, sWAT, and vWAT mass in males and selectively elevated sWAT mass in females (*p_post-weaning diet_* < 0.05; GDM and interaction not significant (n.s.); Figure 1C–E). Similarly, body-fat percentage was raised by post-weaning HFD in both sexes, whereas GDM exposure had no sustained effect (*p_post-weaning diet_* < 0.05; GDM and interaction n.s.; Figure 1F).

At PND21, GDM-exposed offspring of both sexes showed a higher GTT-AUC (*p* < 0.05; Figure 1G). ITT could not be completed in NGT males because their blood glucose fell below the safety limit; among females, GDM exposure elevated glucose levels at every ITT time point and increased the AUC (*p* < 0.05; Figure 1H). By week 9, two-way ANOVA revealed main effects of maternal GDM and post-weaning HFD on both GTT and ITT AUC in both sexes (*p*_post-weaning diet_ < 0.05; *p*_GDM_ < 0.05; *p*_interaction_ < 0.05; Figure 1I–L). Bonferroni post-tests showed impaired glucose tolerance in the NGT-HFD, GDM-chow, and GDM-HFD groups versus NGT-chow (*p* < 0.05; Figure 1I,J), whereas insulin sensitivity declined only in the GDM-HFD group (*p* < 0.05; Figure 1K,L).

### 3.2. Circulating Glucose, Lipids, and CTRP6 Levels in GDM Offspring

**Figure 2:**
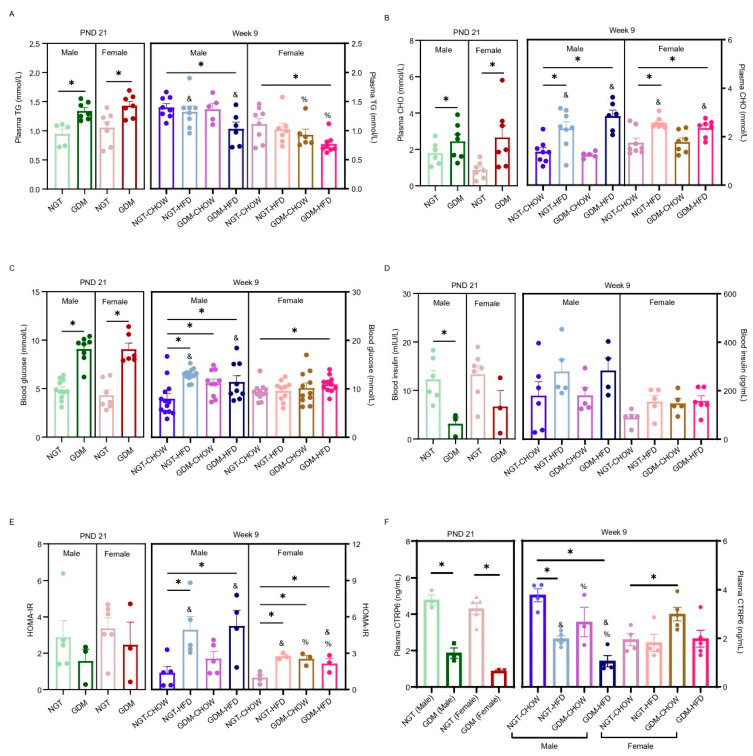
Circulating glucose, lipids, and CTRP6 levels in GDM offspring. (**A**) Plasma triglycerides (TG) levels, *n* = 5–8/group. (**B**) Plasma cholesterol (CHO) levels, *n* = 5–8/group. (**C**) Fasting blood glucose levels, *n* = 6–13/group. (**D**) Fasting plasma insulin levels, *n* = 3–7/group. (**E**) Homeostasis model assessment of insulin resistance (HOMA-IR), *n* = 3–7/group. (**F**) Plasma CTRP6 levels. *n* = 3–5/group. Data are shown as mean ± SEM. Datasets from PND21 were analyzed using an unpaired *t*-test. For the four offspring groups at 9 weeks, a two-way ANOVA was used; where interaction was significant, Bonferroni’s post hoc test was applied. %—main effect of GDM, *p* < 0.05 vs. maternal NGT groups; &—main effect of post-weaning HFD, *p* < 0.05 vs. post-weaning chow groups. * *p* < 0.05, for Bonferroni post-tests. *n* represents the number of litters.

We next profiled circulating CTRP6 alongside glucose and lipid parameters. At weaning, GDM elevated plasma TG in both sexes and CHO selectively in females (*p* < 0.05; Figure 2A,B). Conversely, TG values fell with GDM in females (*p_GDM_* < 0.05) and with post-weaning HFD in males (*p_post-weaning diet_* < 0.05), with significant interactions in both sexes (*p_interaction_* < 0.05; Figure 2A). By week 9, CHO was increased only by post-weaning HFD, whereas GDM had no effect (*p_post-weaning diet_* < 0.05; GDM and interaction n.s., Figure 2B). Fasting blood glucose (FBG) was significantly elevated by GDM exposure at weaning (*p* < 0.05; Figure 2C); this maternal GDM effect disappeared in adult offspring. Thereafter, post-weaning HFD specifically increased FBG in males (*p_post-weaning diet_* < 0.05; *p_interaction_* < 0.05). In females, HOMA-IR was elevated by the combination of GDM and post-weaning HFD (*p_GDM_* < 0.05; *p_post-weaning diet_* < 0.05, *p_interaction_* < 0.05; Figure 2E), whereas in males it was impaired only by maternal GDM (*p_post-weaning diet_* < 0.05; *p_interaction_* < 0.05; Figure 2E). ELISA revealed that GDM exposure significantly lowered plasma CTRP6 in both sexes at weaning (*p* < 0.05; Figure 2F). By week 9, GDM and post-weaning HFD together suppressed CTRP6 in males without interaction (*p_GDM_ <* 0.05; *p_post-weaning diet_* < 0.05; *p_interaction_* > 0.05, Figure 2F), whereas female levels were unchanged.

### 3.3. Morphology Changes in Adipose Tissue in GDM Offspring

**Figure 3:**
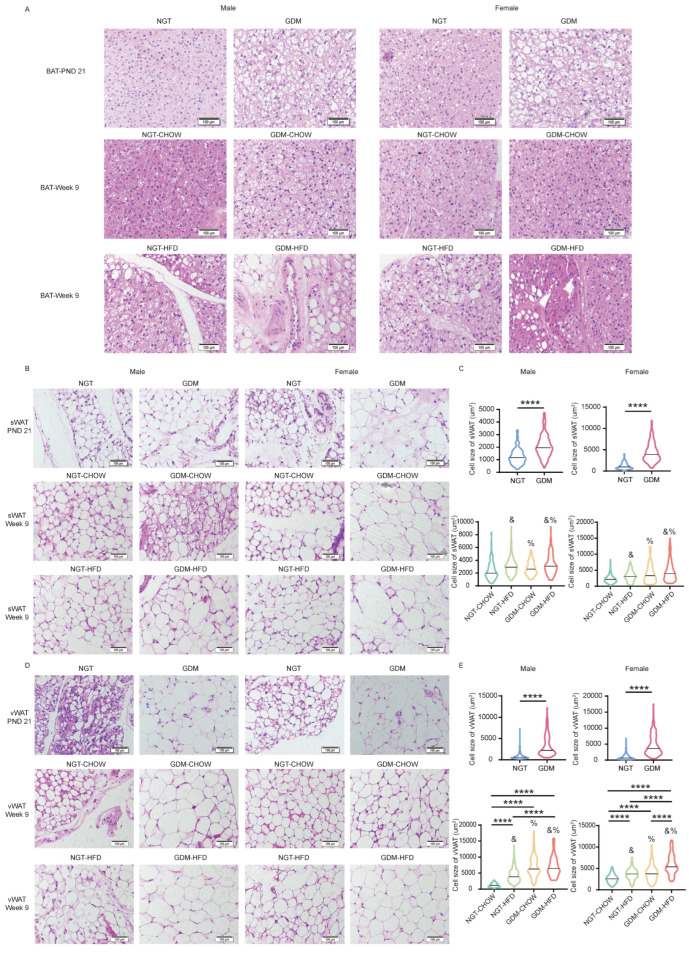
Histological analysis of adipose tissue. (**A**) Hematoxylin and eosin (H&E) staining of iBAT at PND21 and week 9. (**B**,**C**) H&E staining and quantification of adipocyte size in sWAT at PND21 and week 9. (**D**,**E**) H&E staining and quantification of adipocyte size in vWAT at PND21 and week 9. Data were collected from H&E-stained sections of three individual mice in each group, five fields per mouse, 20–40 cells per field, using ImageJ software. Data are shown as mean ± SEM. At PND21, differences between the two groups were evaluated with an unpaired two-tailed *t*-test. For the four offspring groups at 9 weeks, a two-way ANOVA was used; where interaction was significant, Bonferroni’s post hoc test was applied. %—main effect of GDM, *p* < 0.05 vs. maternal NGT groups; &—main effect of post-weaning HFD, *p* < 0.05 vs. post-weaning chow groups. **** *p* < 0.0001, for Bonferroni post-tests. Scale bar, 100 µm.

Consistent with greater iBAT and sWAT mass, GDM induced “whitening” of iBAT and adipocyte hypertrophy in both WAT depots at weaning; mean adipocyte size was larger than in NGT pups (*p* < 0.05; Figure 3A–E). By week 9, two-way ANOVA revealed significant main effects of both maternal GDM and post-weaning HFD on adipocyte hypertrophy in sWAT and vWAT (*p_GDM_* < 0.05, *p_post-weaning diet_* < 0.05; Figure 3B–E), with a significant interaction limited to vWAT (*p_interaction_* < 0.05; Figure 3D,E). Consequently, vWAT adipocytes were enlarged in all three intervention groups relative to NGT-chow controls (*p* < 0.05; Figure 3D,E).

### 3.4. CTRP6 Expression in the iBAT, sWAT, and vWAT

**Figure 4:**
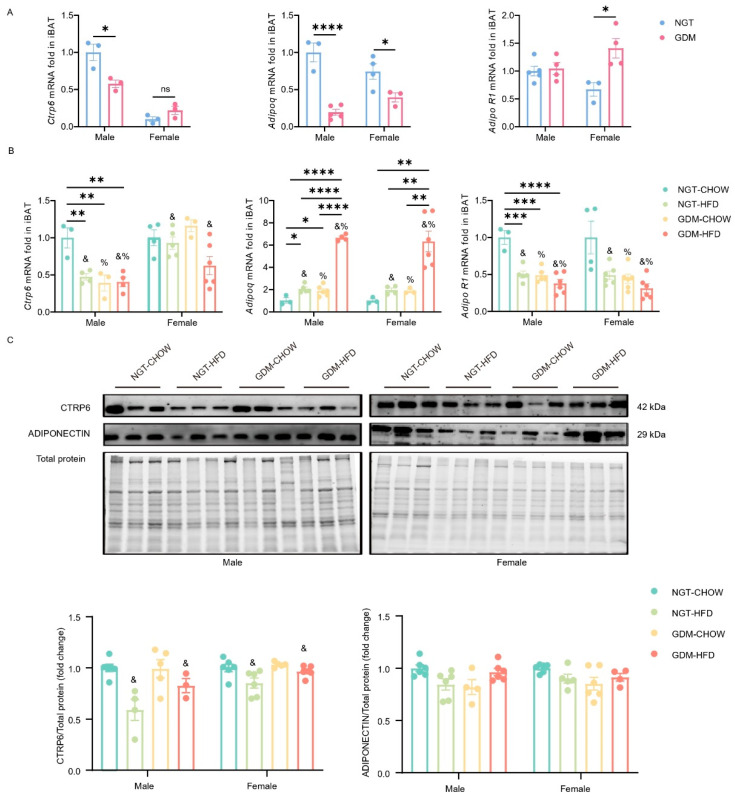
Gene and protein expression of CTRP6 and Adiponectin in iBAT. (**A**) mRNA level of *Ctrp6*, *Adipoq* and *AdipoR1* in iBAT from male and female offspring at PND21, *n* = 3–5/group. (**B**) mRNA level of *Ctrp6*, *Adipoq,* and *AdipoR1* in BAT from male and female offspring at week 9, *n* = 3–6/group. (**C**) Representative Western blotting analyses of CTRP6 and Adiponectin protein in iBAT at week 9, *n* = 4–6/group. Data are shown as mean ± SEM. At PND21, differences between the two groups were evaluated with an unpaired two-tailed *t*-test. For the four offspring groups at 9 weeks, a two-way ANOVA was used; where interaction was significant, Bonferroni’s post hoc test was applied. %—main effect of GDM, *p* < 0.05 vs. maternal NGT groups; &—main effect of post-weaning HFD, *p* < 0.05 vs. post-weaning chow groups. * *p* < 0.05, ** *p* < 0.01, *** *p* < 0.001, **** *p* < 0.0001, for Bonferroni post-tests. *n* represents the number of litters. ns, not significant.

We next quantified CTRP6, Adipoq, and AdipoR1 in three fat depots. At PND21, GDM lowered iBAT *Ctrp6* mRNA in males (*p* < 0.05) and *Adipoq* in both sexes (*p* < 0.05), while raising *AdipoR1* only in females (*p* < 0.05; Figure 4A). By week 9, in males, *Ctrp6* remained suppressed in all intervention groups versus NGT-chow (*p*_GDM_ < 0.05; *p*_post-weaning diet_ < 0.05; *p_interaction_* < 0.05); in females, the reduction was driven mainly by post-weaning HFD (*p_post-weaning diet_* < 0.05; GDM and interaction n.s., Figure 4B). Both maternal GDM and post-weaning HFD increased *Adipoq* but decreased *AdipoR1* mRNA in iBAT (*p_GDM_* < 0.05; *p_post-weaning diet_* < 0.05; *p_interaction_* < 0.05 for *Adipoq* in both sexes and for *AdipoR1* in males; Figure 4B). Consistently, iBAT CTRP6 protein was reduced by post-weaning HFD in both sexes (*p_post-weaning diet_* < 0.05; GDM and interaction n.s.; Figure 4C), whereas adiponectin protein remained unchanged.

**Figure 5:**
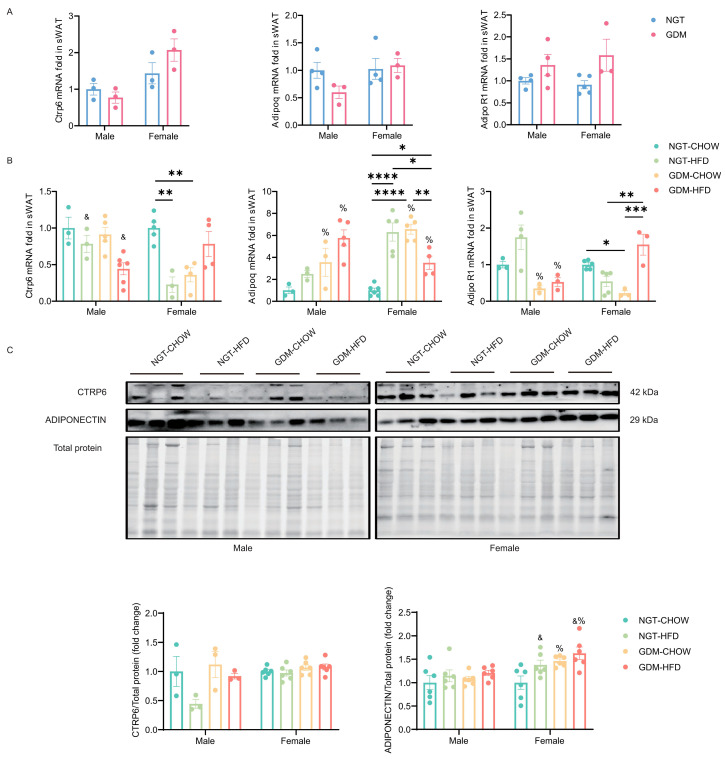
Gene and protein expression of CTRP6 and Adiponectin in sWAT. (**A**) mRNA level of *Ctrp6*, *Adipoq* and *AdipoR1* in sWAT at PND21, *n* = 3–5/group. (**B**) mRNA level of *Ctrp6*, *Adipoq,* and *AdipoR1* in sWAT from male and female offspring at week 9, *n* = 3–6/group. (**C**) Representative Western blotting analyses of CTRP6 and Adiponectin protein in sWAT at week 9, *n* = 3–6/group. Data are shown as mean ± SEM. At PND21, differences between the two groups were evaluated with an unpaired two-tailed *t*-test. For the four offspring groups at 9 weeks, a two-way ANOVA was used; where interaction was significant, Bonferroni’s post hoc test was applied. %—main effect of GDM, *p* < 0.05 vs. maternal NGT groups; &—main effect of post-weaning HFD, *p* < 0.05 vs. post-weaning chow groups. * *p* < 0.05, ** *p* < 0.01, *** *p* < 0.001, **** *p* < 0.0001, for Bonferroni post-tests. *n* represents the number of litters.

At weaning, transcript levels of *Ctrp6*, *Adipoq,* and *AdipoR1* in sWAT did not differ between GDM and NGT pups of either sex (Figure 5A). By week 9, post-weaning HFD had become the dominant regulator. In males, it alone reduced *Ctrp6* mRNA regardless of maternal diet (*p*_post-weaning diet_ < 0.05; GDM and interaction n.s.), whereas in females the same down-regulation was observed in the NGT-HFD and GDM-chow groups (*p_interaction_* < 0.05; main effects n.s.; Figure 5B). Maternal GDM, in contrast, elevated *Adipoq* transcripts in both sexes and suppressed *AdipoR1* in males (*p*_GDM_ < 0.05; *p*_post-weaning diet_ > 0.05; *p_interaction_* < 0.05 for *Adipoq* in females; Figure 5B). Despite these mRNA changes, CTRP6 protein in sWAT at week 9 was unaltered across groups (Figure 5C), whereas adiponectin protein was consistently up-regulated by GDM and post-weaning HFD independently (*p*_GDM_ < 0.05; *p*_post-weaning diet_ < 0.05; interaction n.s.; Figure 5C).

**Figure 6:**
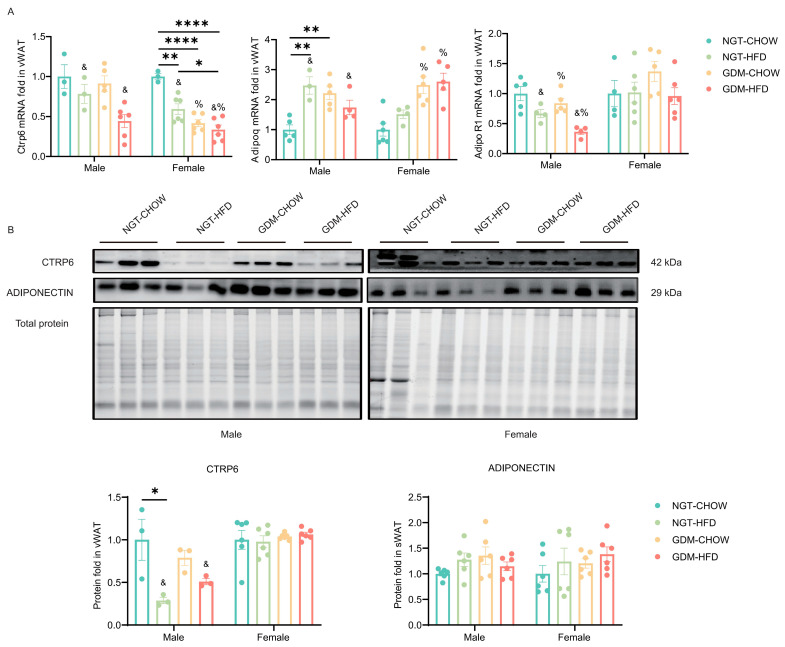
Gene and protein expression of CTRP6 and Adiponectin in vWAT. (**A**) mRNA level of *Ctrp6*, *Adipoq*, and *AdipoR1* in vWAT at week 9, *n* = 3–6/group. (**B**) Representative Western blotting analyses of CTRP6 and Adiponectin protein in vWAT at week 9, *n* = 3–6/group. Data are shown as mean ± SEM. Data were analyzed by a two-way ANOVA first, with Bonferroni’s multiple comparisons test if an interaction was present. %—main effect of GDM, *p* < 0.05 vs. maternal NGT groups; &—main effect of post-weaning HFD, *p* < 0.05 vs. post-weaning chow groups. * *p* < 0.05, ** *p* < 0.01, **** *p* < 0.0001, for Bonferroni post-tests. *n* represents the number of litters.

Due to the limited mass of vWAT at weaning, molecular analyses were restricted to week-9 offspring. In males, post-weaning HFD alone sufficed to reduce *Ctrp6* mRNA (*p_post-weaning diet_* < 0.05; GDM and interaction n.s.), whereas in females *Ctrp6* was lower in every intervention group than in the NGT-chow group, with main effects of both GDM and post-weaning HFD plus a significant interaction (*p*_post-weaning diet_ < 0.05, *p*_GDM_ < 0.05; *p_interaction_* < 0.05; Figure 6A). *Adipoq* mRNA was up-regulated by post-weaning HFD in males (*p*_post-weaning diet_ < 0.05; *p_interaction_* < 0.05; Figure 6A) and by GDM in females (*p*_GDM_ < 0.05, post-weaning HFD, and interaction n.s., Figure 6A). *AdipoR1* expression was lowered by both GDM exposure and post-weaning HFD (*p*_GDM_ < 0.05; *p*_post-weaning diet_ < 0.05; *p_interaction_* > 0.05) but remained unchanged in females (Figure 6A). Protein data only partially mirrored RNA: CTRP6 was reduced in NGT-HFD males by post-weaning HFD (*p*_post-weaning diet_ < 0.05, *p*_GDM_ > 0.05, *p_interaction_* < 0.05), whereas female CTRP6 and adiponectin protein levels were unaltered across groups (Figure 6B).

## 4. Discussion

We provide the first longitudinal map of CTRP6 across interscapular brown (iBAT), subcutaneous white (sWAT), and visceral white (vWAT) adipose depots in male and female offspring exposed to maternal GDM. GDM offspring exhibited increased adiposity and impaired glucose tolerance at weaning; although adiposity normalized by adulthood, glucose intolerance persisted and was exacerbated by a post-weaning HFD. Reduced plasma CTRP6 was detected in both sexes at weaning, accompanied by depot-specific suppression of CTRP6 protein in male iBAT. Into adulthood, males maintained low circulating and iBAT CTRP6, whereas females rebounded to NGT control levels when maintained on chow, a pattern that coincided with coordinated up-regulation of adiponectin. These findings position CTRP6 as an early, sex-biased marker of GDM-induced metabolic programming rather than a passive consequence of obesity.

GDM offspring displayed higher body weight, iBAT, and sWAT mass, and fat percentage at weaning. This neonatal excess adiposity had disappeared by adulthood, consistent with rodent [26,27] and human [28,29] studies linking maternal glycemia to early fat accretion [30,31,32]. Prior work shows that maternal hyperglycemia programs metabolism differently in the two sexes: in humans, GDM-linked obesity risk emerges primarily in boys [33,34], yet glucose dysregulation is more pronounced in girls [33,35], whereas rodent studies report that females are more vulnerable to disturbances in glucose homeostasis and males to increased adiposity [36,37]. Consistently, our data replicate this sex-specific pattern: although glucose and insulin tolerance were impaired in both males and females at weaning and in young adulthood, HOMA-IR remained unchanged in newly weaned pups yet rose significantly in adults. Crucially, the adult increase in HOMA-IR was driven mainly by female offspring, with no comparable effect in males. Although GDM is linked to childhood dyslipidemia [38], its adult impact is unclear. We observed elevated TG and CHO at weaning in both sexes, yet GDM did not affect adult CHO; instead, post-weaning HFD raised CHO. Surprisingly, HFD lowered TG in males, whereas GDM lowered it in females. Adiponectin is well recognized for lowering plasma triglycerides and protecting against atherosclerosis [39,40]. GDM increased adiponectin expression in all three depots at week 9; together with enlarged sWAT/vWAT adipocytes, this may represent a compensatory mechanism that reduces plasma TG in GDM-exposed adult offspring.

Unlike adiponectin, which protects against insulin resistance, diabetes, and atherosclerosis, CTRP6 functions as a pro-inflammatory factor and an adipogenesis inhibitor, thereby impairing insulin sensitivity [18]. Adipose tissue is the primary source of CTRP6, and its function in adipose tissue expansion, inflammation, and nutrient sensing has been well documented in several studies [16,18,23,41,42]. At weaning, plasma CTRP6 decreased in both sexes, but only male pups showed reduced levels in iBAT—not sWAT—indicating iBAT is a key source of circulating CTRP6. This iBAT-specific suppression persisted into adulthood in males. In contrast, females on a chow diet had higher plasma CTRP6 despite lower vWAT expression, implying extra-adipose, hormone-sensitive production, since CTRP6 is also highly expressed in the endometrium in females [21]. CTRP6 knockdown has been shown to protect against diet-induced obesity by up-regulating brown-fat markers and mitochondrial metabolic factors [20]. Thus, low iBAT CTRP6 in males and low vWAT CTRP6 in females could contribute to sex-specific metabolic phenotypes. Identifying the still-uncharacterized CTRP6 receptor and elucidating its signaling cascade will clarify how these depot- and sex-biased alterations translate into long-term metabolic risk.

Our results differ from earlier reports that found elevated CTRP6 levels in plasma and adipose tissue of obese, type 2 diabetic humans and mice, which correlated positively with BMI, waist-hip ratio, and markers of insulin resistance [18,19]. Short-term HFD also rapidly up-regulates adipose CTRP6 in mice, an effect that precedes inflammatory markers and indicates CTRP6 is sensitive to energy balance [23]. The discrepancy with previous studies suggests two non-exclusive explanations: (1) the reduced plasma TG and increased adiponectin across three adipose depots in our model may reflect a compensatory metabolic adaptation phase; (2) low CTRP6 expression could itself be an adaptive response to HFD, since gain- and loss-of-function studies indicate CTRP6 promotes inflammatory cascades while suppressing adipogenesis and adipose expansion [23]. As an adiponectin paralog, CTRP6 must be considered alongside adiponectin and its receptor signaling. In adult female offspring, GDM exposure elevated adiponectin protein levels in sWAT. This increase, together with reduced CTRP6, points to a coordinated compensatory response in adipose tissue after GDM exposure, consistent with the previously reported inverse regulation of the two adipokines [17,19]. Temporally, AdipoR1 expression in iBAT rose in newly weaned offspring but declined by week 9, indicating a shift from compensation to decompensation during the six-week post-weaning HFD challenge. We therefore propose that decreased CTRP6, combined with elevated adiponectin, may act in concert with the reduction in AdipoR1 signaling in adult offspring.

Nevertheless, our study has several limitations. First, GDM was induced by high-fat feeding, so pure glycemic effects are confounded by maternal obesity and lipid excess; isocaloric or euglycemic-hyperinsulinemic clamp studies are needed. Second, limited tissue mass restricted some Western blot analyses to 3–5 litters; nevertheless, litter-based statistics and Bonferroni correction were applied to minimize type I error. Third, the absence of CTRP6 gain- or loss-of-function experiments precludes causal inference; adipocyte-specific CRISPR or AAV rescue models are now required.

In conclusion, maternal GDM induces sex- and depot-specific CTRP6 alterations linked to offspring metabolic dysfunction. Although weanling offspring exhibited increased adiposity, lipids, and glucose intolerance, only glucose/insulin intolerance persisted into adulthood. Circulating CTRP6 was low at weaning in both sexes but showed a sex-divergent pattern in adults (low in males, high in females). Tissue-specifically, GDM primarily affected iBAT CTRP6 in males and vWAT CTRP6 in females. Future work should decipher the mechanisms behind these distinct remodeling patterns.

## Data Availability

The original contributions presented in the study are included in the Supplementary Material, File S1; further inquiries can be directed to the corresponding author.

## Supplementary Materials

The following supporting information can be downloaded at: https://www.mdpi.com/article/10.3390/biomedicines14010224/s1.
